# Supplementary material for: Optical Properties Investigation of Upconverting K2Gd(PO4)(WO4):20%Yb3+,Tm3+ Phosphors
Source: Materials (Basel). 2023 Feb 3;16(3):1305. doi: 10.3390/ma16031305 (PMC9920437; doi:10.3390/ma16031305)
Supplement: Supplementary file 1 [file materials-16-01305-s001.zip › materials-2148984-supplementary.pdf]

materials

Supplementary Materials

Optical properties investigation of upconverting $K_2Gd(PO_4)(WO_4)$:20%Yb^{3+},Tm^{3+} phosphors

Julija Grigorjevaite * and Arturas Katelnikovas *

Institute of Chemistry, Faculty of Chemistry and Geosciences, Vilnius University, Naugarduko 24, LT–03225 Vilnius, Lithuania
* Correspondence: julija.grigorjevaite@chf.vu.lt (J.G.); arturas.katelnikovas@chf.vu.lt (A.K.)

Table S1. Spectrometer settings for measuring reflection spectra of KGPW:20%Yb^{3+},Tm^{3+} phosphors.

**KGPW:20%Yb^{3+},Tm^{3+}**	
**Parameter**	
EmBW	0.15 nm
ExBW	4.00 nm
Step	0.50 nm
Integration time	0.200 s
Range	250 – 800 nm
Repeats	3

Citation: Grigorjevaite, J.; Katelnikovas, A. Optical Properties Investigation of Upconverting $K_2Gd(PO_4)(WO_4)$:20%Yb^{3+},Tm^{3+} Phosphors. *Materials* **2023**, *16*, 1305. https://doi.org/10.3390/ma16031305

Academic Editor: Yuta Matsushima

Received: 23 December 2022
Revised: 22 January 2023
Accepted: 1 February 2023
Published: 3 February 2023

Copyright: © 2023 by the authors. Submitted for possible open access publication under the terms and conditions of the Creative Commons Attribution (CC BY) license (https://creativecommons.org/licenses/by/4.0/).

Table S2. Spectrometer settings for measuring excitation spectra of KGPW:20%Yb^{3+},Tm^{3+} phosphors.

KGPW:20%Yb^{3+},Tm^{3+}		
**Excitation scan**	**Parameter**	
λ_{em} = 450 nm	EmBW	1.60 nm
	ExBW	0.50 nm
	Dwell	0.200 s
	Step	0.50 nm

Table S3. Spectrometer settings for measuring DC emission spectra of KGPW:20%Yb^{3+},Tm^{3+} phosphors.

KGPW:20%Yb^{3+},Tm^{3+}		
**Emission scan**	**Parameter**	
λ_{ex} = 360 nm	EmBW	0.50 nm
	ExBW	1.60 nm
	Dwell	0.200 s
	Step	0.50 nm

Table S4. Spectrometer settings for measuring UC emission spectra of KGPW:20%Yb^{3+},Tm^{3+} phosphors.

KGPW:20%Yb^{3+},Tm^{3+}		
**Emission scan**	**Parameter**	
λ_{ex} = 980 nm	EmBW	0.06 nm
	ExBW	N/A
	Dwell	0.200 s
	Step	0.50 nm

Table S5. Lattice parameters of KGPW, KGPW:5%Tm^{3+}, KGPW:20%Yb^{3+}, and KGPW:20%Yb^{3+},5%Ho^{3+} samples derived from Rietveld refinement analysis.

**Sample**	***a*, Å**	***b*, Å**	***c*, Å**	***V*, Å^3**	**Ref.**
$K_2Ho(PO_4)(WO_4)$	6.8820	12.1485	19.6950	1646.6	**[1]**
$K_2Gd(PO_4)(WO_4)$	6.94294	12.24594	19.68550	1673.7	This work
$K_2Gd(PO_4)(WO_4)$:5%Tm^{3+}	6.94232	12.24028	19.69242	1673.4	This work
$K_2Gd(PO_4)(WO_4)$:20%Yb^{3+}	6.92142	12.20985	19.67467	1662.7	This work
$K_2Gd(PO_4)(WO_4)$:20%Yb^{3+}, 5%Tm^{3+}	6.92048	12.20600	19.67702	1662.1	This work

Table S6. Color coordinates (CIE 1931 color space) of KGPW:Tm^{3+} and KGPW:20%Yb^{3+},Tm^{3+} as a function of Tm^{3+} concentration and excitation wavelength.

**Tm^{3+} (%)**	**KGPW:Tm^{3+}**		**KGPW:20%Yb^{3+},Tm^{3+}**			
	**λ_{ex} = 360 nm**		**λ_{ex} = 360 nm**		**λ_{ex} = 980 nm**	
	**x**	**y**	**x**	**y**	**x**	**y**
0.5	0.15762	0.02803	0.15797	0.03672	0.14463	0.09657
1	0.15763	0.02889	0.15772	0.02931	0.16312	0.10567
2	0.15772	0.02840	0.15738	0.02821	0.14411	0.09332
5	0.15735	0.02924	0.15709	0.02718	0.14628	0.09301

Table S7. UC PL rise time and lifetime values of KGPW:Tm^{3+} and KGPW:20%Yb^{3+},Tm^{3+} phosphors as a function of Tm^{3+} concentration, emission wavelength, and excitation wavelength.

	**Rise time (μs)**	**Std. dev. (μs)**	**Lifetime τ_{eff} (μs)**	**Std. dev. (μs)**
**Tm^{3+}(%)**	**λ_{ex} = 360 nm λ_{em} = 450 nm**			
	**KGPW:Tm^{3+}**			
0.5	-	-	24	1
1	-	-	34	1
2	-	-	23	1
5	-	-	20	1
**Tm^{3+}(%)**	**λ_{ex} = 360 nm λ_{em} = 450 nm**			
	**KGPW:20%Yb^{3+},Tm^{3+}**			
0.5	-	-	22	1
1	-	-	22	1
2	-	-	21	1
5	-	-	19	1
**Tm^{3+}(%)**	**λ_{ex} = 980 nm λ_{em} = 478 nm**			
	**KGPW:20%Yb^{3+},Tm^{3+}**			
0.5	73	8	201	5
1	68	8	159	2
2	63	7	138	2
5	38	3	107	1
**Tm^{3+}(%)**	**λ_{ex} = 980 nm λ_{em} = 800 nm**			
	**KGPW:20%Yb^{3+},Tm^{3+}**			
0.5	36	2	267	3
1	52	6	204	3
2	56	7	158	2
5	39	2	101	1

Table S8. The calculated PL lifetime values (λ_{ex} = 980 nm, λ_{em} = 1050 nm) of Yb^{3+} emission in KGPW:20%Yb^{3+},Tm^{3+}, and $Yb^{3+} \rightarrow Tm^{3+}$ energy transfer efficiency (η_{tr}) as a function of Tm^{3+} concentration.

	**Lifetime τ_{eff} (μs)**	**Std. dev. (μs)**	**η_{tr} (%)**
**Tm^{3+}(%)**	**KGPW:20%Yb^{3+},Tm^{3+}**		
0	1312	24	-
0.5	738	5	44
1	544	4	59
2	457	4	65
5	416	4	68

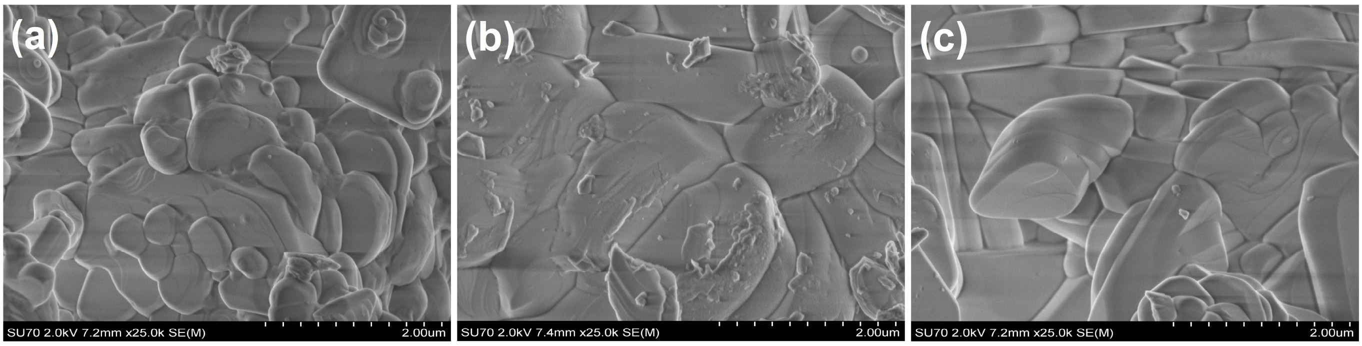

Figure S1. SEM images of (**a**) KGPW:5%Yb^{3+},(**b**) KGPW:20%Yb^{3+}, and (**c**) KGPW:20%Yb^{3+},5%Tm^{3+}.

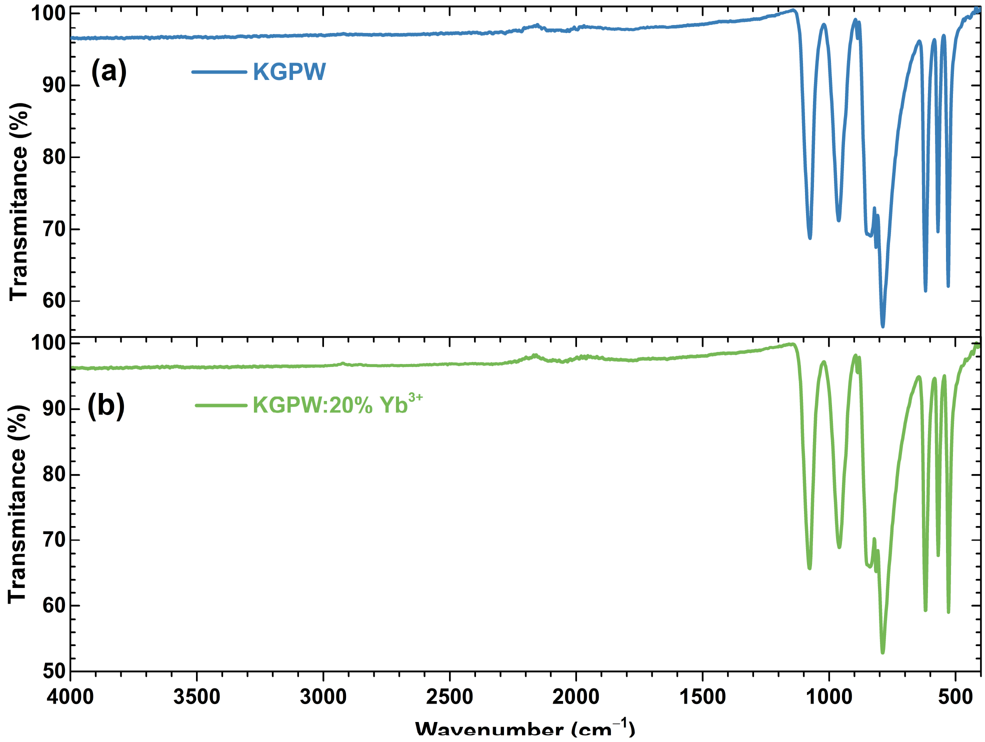

Figure S2. IR spectra of (**a**) undoped KGPW and (**b**) KGPW:20%Yb^{3+}.

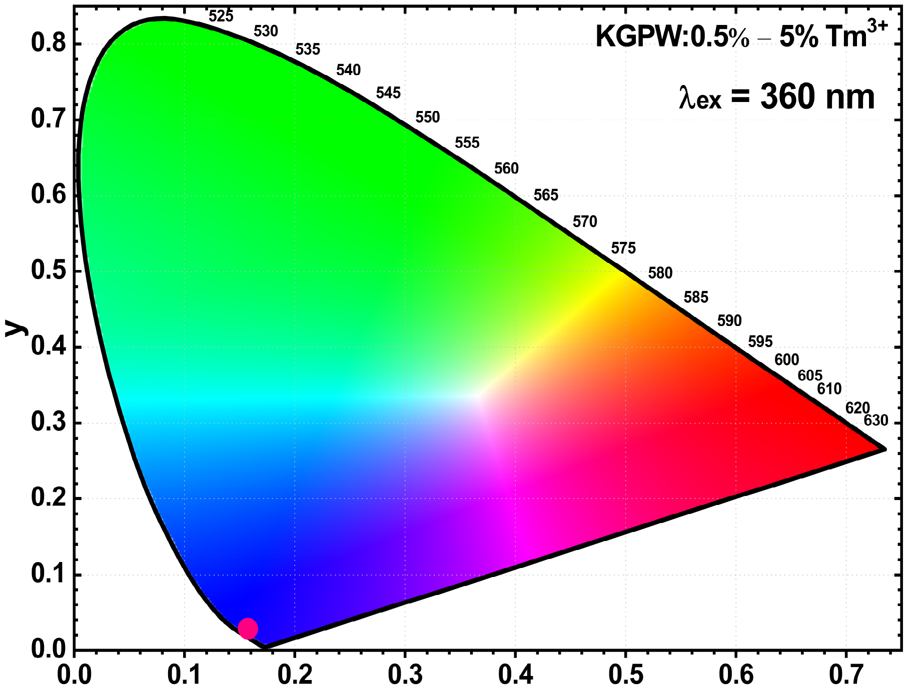

Figure S3. CIE 1931 color space diagram and color coordinates of KGPW:Tm^{3+} as a function of Tm^{3+} concentration under 360 nm excitation.
